# The Spectrum of COVID-19-Associated Myocarditis: A Patient-Tailored Multidisciplinary Approach

**DOI:** 10.3390/jcm10091974

**Published:** 2021-05-04

**Authors:** Giovanni Peretto, Andrea Villatore, Stefania Rizzo, Antonio Esposito, Giacomo De Luca, Anna Palmisano, Davide Vignale, Alberto Maria Cappelletti, Moreno Tresoldi, Corrado Campochiaro, Silvia Sartorelli, Marco Ripa, Monica De Gaspari, Elena Busnardo, Paola Ferro, Maria Grazia Calabrò, Evgeny Fominskiy, Fabrizio Monaco, Giulio Cavalli, Luigi Gianolli, Francesco De Cobelli, Alberto Margonato, Lorenzo Dagna, Mara Scandroglio, Paolo Guido Camici, Patrizio Mazzone, Paolo Della Bella, Cristina Basso, Simone Sala

**Affiliations:** 1 Department of Cardiac Electrophysiology and Arrhythmology, IRCCS San Raffaele Scientific Institute, 20132 Milan, Italy; mazzone.patrizio@hsr.it (P.M.); dellabella.paolo@hsr.it (P.D.B.); sala.simone@hsr.it (S.S.); 2 Myocarditis Disease Unit, IRCCS San Raffaele Scientific Institute, 20132 Milan, Italy; esposito.antonio@unisr.it (A.E.); deluca.giacomo@hsr.it (G.D.L.); palmisano.anna@hsr.it (A.P.); vignale.davide@hsr.it (D.V.); campochiaro.corrado@hsr.it (C.C.); sartorelli.silvia@hsr.it (S.S.); busnardo.elena@hsr.it (E.B.); 3 School of Medicine, San Raffaele Vita-Salute University, 20132 Milan, Italy; a.villatore@studenti.unisr.it (A.V.); decobelli.francesco@hsr.it (F.D.C.); dagna.lorenzo@unisr.it (L.D.); camici.paolo@hsr.it (P.G.C.); 4 Department of Cardiac Thoracic Vascular Sciences and Public Health, Cardiovascular Pathology, Padua University, 35128 Padua, Italy; s.rizzo@unipd.it (S.R.); monica.deg1@gmail.com (M.D.G.); cristina.basso@unipd.it (C.B.); 5 Experimental Imaging Center, Radiology Unit, IRCCS San Raffaele Scientific Institute, 20132 Milan, Italy; 6 Unit of Immunology, Rheumatology, Allergy and Rare Diseases (UnIRAR), IRCCS San Raffaele Scientific Institute, 20132 Milan, Italy; cavalli.giulio@hsr.it; 7 Department of Clinical Cardiology and Intensive Care Unit, IRCCS San Raffaele Scientific Institute, 20132 Milan, Italy; cappelletti.alberto@hsr.it (A.M.C.); margonato.alberto@hsr.it (A.M.); 8 Unit of General Medicine and Advanced Care, IRCCS San Raffaele Scientific Institute, 20132 Milan, Italy; tresoldi.moreno@hsr.it; 9 COVID-19 Unit, IRCCS San Raffaele Scientific Institute, 20132 Milan, Italy; ripa.marco@hsr.it; 10 Department of Infectious Diseases, IRCCS San Raffaele Scientific Institute, 20132 Milan, Italy; 11 Department of Nuclear Medicine, IRCCS San Raffaele Scientific Institute, 20132 Milan, Italy; ferro.paola@hsr.it (P.F.); gianolli.luigi@hsr.it (L.G.); 12 Cardiac Surgery Intensive Care Unit, Division of Anesthesiology, IRCCS San Raffaele Scientific Institute, 20132 Milan, Italy; calabro.mariagrazia@hsr.it (M.G.C.); fominskiy.evgeny@hsr.it (E.F.); monaco.fabrizio@hsr.it (F.M.); scandroglio.mara@hsr.it (M.S.)

**Keywords:** myocarditis, COVID-19, SARS-CoV-2, ventricular arrhythmias, endomyocardial biopsy, cardiac magnetic resonance, multidisciplinary, immunosuppression, inflammation

## Abstract

Background. Myocarditis lacks systematic characterization in COVID-19 patients. Methods. We enrolled consecutive patients with newly diagnosed myocarditis in the context of COVID-19 infection. Diagnostic and treatment strategies were driven by a dedicated multidisciplinary disease unit for myocarditis. Multimodal outcomes were assessed during prospective follow-up. Results. Seven consecutive patients (57% males, age 51 ± 9 y) with acute COVID-19 infection received a de novo diagnosis of myocarditis. Endomyocardial biopsy was of choice in hemodynamically unstable patients (*n* = 4, mean left ventricular ejection fraction (LVEF) 25 ± 9%), whereas cardiac magnetic resonance constituted the first exam in stable patients (*n* = 3, mean LVEF 48 ± 10%). Polymerase chain reaction (PCR) analysis revealed an intra-myocardial SARS-CoV-2 genome in one of the six cases undergoing biopsy: in the remaining patients, myocarditis was either due to other viruses (*n* = 2) or virus-negative (*n* = 3). Hemodynamic support was needed for four unstable patients (57%), whereas a cardiac device implant was chosen in two of four cases showing ventricular arrhythmias. Medical treatment included immunosuppression (43%) and biological therapy (29%). By the 6-month median follow-up, no patient died or experienced malignant arrhythmias. However, two cases (29%) were screened for heart transplantation. Conclusions. Myocarditis associated with acute COVID-19 infection is a spectrum of clinical manifestations and underlying etiologies. A multidisciplinary approach is the cornerstone for tailored management.

## 1. Introduction

Among a wide range of cardiovascular manifestations, myocarditis is a possible complication of the severe acute respiratory syndrome coronavirus-2 (SARS-CoV-2) infection responsible for the current COVID-19 pandemic [1,2]. Due to the lack of investigation by endomyocardial biopsy (EMB) and cardiac magnetic resonance (CMR), however, in many published reports, myocarditis was just clinically suspected and largely undistinguishable from other causes of myocardial injury [1,3]. In turn, treatment strategies and outcomes of COVID-19-associated myocarditis are still to be defined. We report the first series of patients diagnosed with acute SARS-CoV-2 infection undergoing advanced diagnostic characterization and multidisciplinary management for associated myocarditis, proven by gold standard diagnostic techniques. In particular, we aimed at describing the wide spectrum of COVID-19-associated myocarditis, and the results of a patient-tailored management by a dedicated “myocarditis disease unit” [4].

## 2. Materials and Methods

Consecutive patients with clinically suspected myocarditis in the context of acute SARS-CoV-2 infection were enrolled from March 2020 to December 2020 at a third-level referral center for COVID-19 management. As a part of an internal protocol, approved by the local institutional review board, written informed consent was obtained from all patients. Diagnostic and therapeutic workup and uniform study endpoints are reported in Online Supplements.

## 3. Results

Seven consecutive patients (0.2% of the COVID-19 inpatients during the enrollment period) were diagnosed with myocarditis. Clinical features of every single case are presented in the following section. Baseline characteristics of the whole series are summarized in Table 1, whereas treatment strategies and study endpoints are reported in Table 2.

### 3.1. Patient 1 (P1)

**Figure 1:**
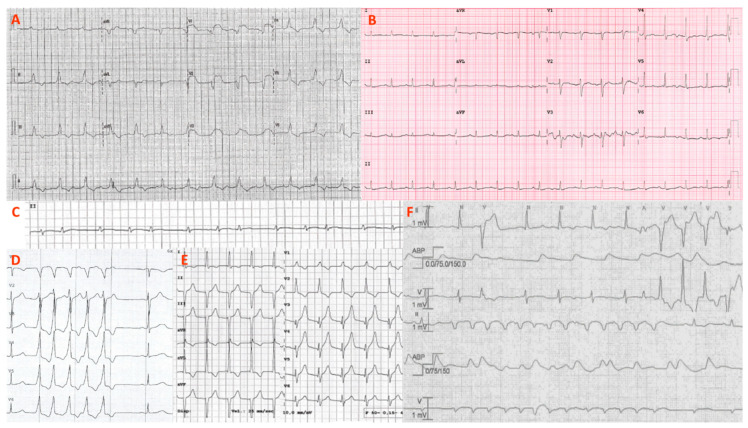
ECG and arrhythmias. Representative ECGs and arrhythmias from the patient series (P1–P7) are shown. (**A**) Atrioventricular dissociation with huge anterior ST elevation (P7); (**B**) sinus tachycardia with low QRS voltages and diffuse repolarization abnormalities (P2); (**C**) paroxysmal atrial fibrillation detected by ICU telemonitoring (P7); (**D**) nonsustained ventricular tachycardia (P4); (**E**) transitory, self-limited, accelerated junctional rhythm (P1); (**F**) polymorphic and irregular nonsustained ventricular tachycardias during ICU stay (P5). ICU = intensive care unit.

A 43-year-old woman was admitted to hospital for epigastric pain. The nasopharyngeal swab (NPS) was positive for SARS-CoV-2, and the computed tomography (CT) scan showed bilateral pneumonia. The electrocardiogram (ECG) had mild ST-segment elevation and transitory phases of an accelerated junctional rhythm (Figure 1). The left ventricular ejection fraction (LVEF) was 43%, and both T-troponin (T-Tn) and NTproBNP were elevated. Coronary artery disease was ruled out by CT scan. Cardiac magnetic resonance (CMR) showed diffuse edema and a reverse takotsubo kinetic pattern. Endomyocardial biopsy (EMB) was performed at bedside and showed virus-negative lymphocytic myocarditis with minimal necrosis. Serum anti-heart autoantibodies (AHA) tested positive. Since early recovery was observed (final LVEF 65%, absent arrhythmias, normal T-Tn), the patient was discharged with no therapy. The nine-month follow-up was uneventful.

### 3.2. Patient 2 (P2) 

A 38-year-old man was symptomatic for cough and dyspnea, but afebrile. NPS and CT were consistent with COVID-19 interstitial pneumonia. The ECG was notable for diffuse T-wave abnormalities, and the echocardiogram revealed an LVEF of 41% with septal hypokinesia and no chamber dilation. Since the T-Tn curve was unremarkable, coronary arteries were not evaluated. CMR was negative for updated Lake Louise criteria (LLC) [5] but showed septal edema. EMB documented remarkable CD68+ macrophage infiltrates, interstitial edema and spotty fibrosis. The N gene of SARS-CoV-2 was amplified from myocardial tissue, as well as low-load (<500 copies of DNA/µg) parvovirus B19 (PV-B19) in the absence of viremia. The patient was discharged with no etiology-specific treatment. Telemedicine reassessment revealed no symptoms and an LVEF of 63% by the 6-month follow-up.

### 3.3. Patient 3 (P3)

**Figure 2:**
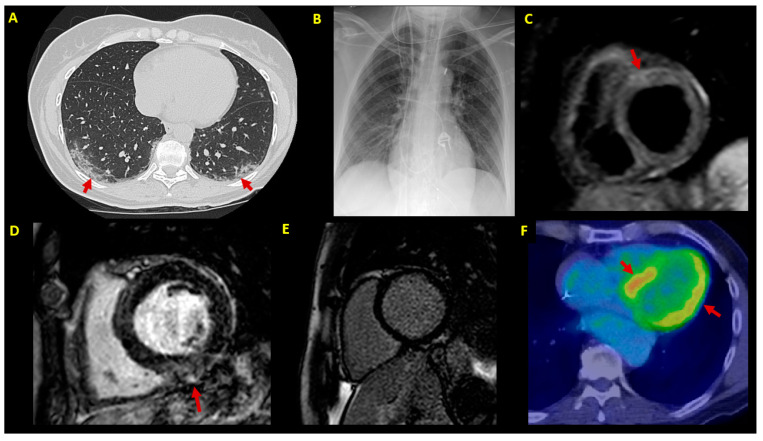
Imaging findings. Imaging findings at patient (P1–P7) diagnostic workup. (**A**) Chest CT scan showing bilateral patchy ground-glass opacities (arrows) (P3); (**B**) chest X-ray in a patient (P7) with cardiogenic shock supported by IABP, VA-ECMO and temporary pacemaker; (**C**) CMR in a patient with infarct-like acute myocarditis associated with COVID-19 (P3); T2-STIR sequence shows edema in the anterior basal segment (arrow); (**D**) LGE sequences in a patient (P5) showing mild inferior mid-myocardial/subepicardial LGE (arrow); (**E**) absence of LGE by 3-month follow-up CMR in a patient (P4) with fulminant myocarditis at presentation; (**F**) 3-month follow-up FDG-PET scan in an ICD carrier (P5) with virus-negative myocarditis; abnormal left ventricular FDG uptake is shown (arrows). CMR = cardiac magnetic resonance; CT = computed tomography; EMB = endomyocardial biopsy; FDG-PET= 18F-fluorodeoxyglucose positron emission tomography; IABP = intra-aortic balloon pump; ICD = implantable cardioverter defibrillator; LGE = late gadolinium enhancement; LVEF = left ventricular ejection fraction; STIR = short-tau inversion recovery; VA-ECMO = venoarterial extracorporeal membrane oxygenator.

A 58-year-old woman presented with acute chest pain after 1 week of fever up to 38.5 °C, vomit and diarrhea. She had no fever (37.0 °C), but the NPS tested positive for COVID-19. The ECG showed inferolateral ST-segment depression with an infarct-like T-Tn curve. The echocardiogram was unremarkable, with an LVEF of 59%. The CT scan both excluded critical coronary stenosis and showed ground-glass lung consolidations, confirming COVID-19 pneumonia. CMR (Figure 2) revealed mild edema and anteroseptal late gadolinium enhancement (LGE), fulfilling criteria for acute myocarditis. On account of clinical stability and T-Tn normalization, EMB was not performed. At the 3-month follow-up, the patient had no chest pain and showed a normal echocardiogram and T-Tn. However, she was still symptomatic for dyspnea on effort, with desaturation up to 86% at the rapid pace walk test.

### 3.4. Patient 4 (P4)

A 45-year-old man was admitted at another institution for epigastric pain. CT showed pneumonia, and the NPS was positive for SARS-CoV-2. The echocardiogram showed an LVEF of 34% with severe biventricular dilation. The coronary CT scan was normal. Due to subsequent hemodynamic instability, the patient was transferred to our intensive care unit (ICU). HD inotropes and intra-aortic balloon pump (IABP) were necessary for 6 days. The NT-proBNP peak was 24,252 pg/mL. As the first-choice exam, EMB showed edema and myocardial inflammation. PCR analysis was negative for SARS-CoV-2, but positive for high-load PV-B19 DNA (>500 copies of DNA/µg). An off-label trial of daily anakinra was started. Due to premature ventricular complexes (PVCs) and nonsustained ventricular tachycardia (NSVT), the patient was discharged on metoprolol. By the 3-month follow-up, T-Tn, NTproBNP and Holter ECG were all normal. CMR showed incomplete systolic function recovery (LVEF 47%) and mildly increased T2 mapping, with no LGE.

### 3.5. Patient 5 (P5)

A 64-year-old male patient was admitted to our ICU following out-of-hospital cardiac arrest secondary to ventricular fibrillation. The ECG showed anterior ST elevation and the LVEF was 15%. After immediate support by invasive mechanical ventilation and HD inotropes, urgent coronary angiography showed absence of culprit lesions. The lung CT scan was unremarkable, and COVID-19 was diagnosed only by repeated NPS. EMB showed virus-negative inflammatory cardiomyopathy. A few days later, CMR showed LLC-proven myocarditis. Telemetry showed multiple episodes of irregular NSVT. Due to family history of sudden cardiac death, the patient underwent genotyping and dual-chamber implantable cardioverter defibrillator (ICD) placement. Furthermore, since abnormal myocardial glucose uptake was described at 3-month 18F-fluorodeoxyglucose positron emission tomography (FDG-PET), oral immunosuppressive therapy was planned but delayed because of evidence of latent tuberculosis at eligibility screening. The indication was finally withheld, since the LVEF improved up to 60% by month 6 on ACE inhibitors. ICD telemetry was uneventful.

### 3.6. Patient 6 (P6)

A 50-year-old man was admitted to an external hospital for acute dyspnea and diagnosed with COVID-19 interstitial pneumonia. The ECG showed sinus tachycardia, first-degree atrioventricular block (AVB) and low QRS voltages. At the echocardiogram, the patient had an LVEF of 20% in the absence of hemodynamic instability. T-Tn was mildly abnormal and NT-proBNP was 1122 pg/mL. Coronary arteries were angiographically normal. Following multiple NSVT episodes, the patient underwent subcutaneous ICD (S-ICD) implant. Since symptomatic LV systolic dysfunction persisted at the 1-month follow-up (LVEF 19%), elective admission on COVID-free status was planned at our institution. EMB documented virus-negative chronically active inflammatory cardiomyopathy. An immunosuppressive therapy (IST) regimen was started. However, the 6-month LVEF was 24% and the patient had persistent dyspnea. Since a preexisting dilated cardiomyopathy was suspected, the patient was both proposed for genetic testing and screened for heart transplantation. 

### 3.7. Patient 7 (P7)

**Figure 3:**
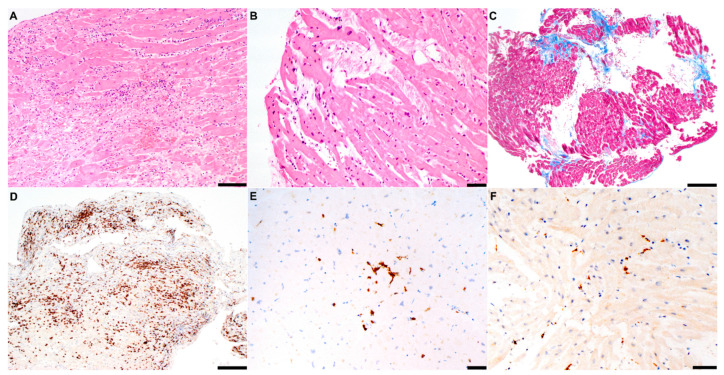
Histology findings. Representative findings at histologic analysis of patients (P1–P7) are shown. (**A**) Hematoxylin-eosin assay in a patient (P7) with fulminant EBV myocarditis complicating COVID-19 infection; diffuse areas of necrosis and massive lymphocytic inflammatory infiltrates are shown; (**B**) mild lymphocytic infiltration without significant necrosis in a patient (P1) with borderline myocarditis; (**C**) trichrome assay in a patient (P6) with myocarditis (not shown) and likely preexisting dilated cardiomyopathy; multiple areas of replacement fibrosis (blue) are shown; (**D**) immunohistochemistry for CD3+ T-lymphocytes (>7/mm^2^) in fulminant myocarditis (P7); (**E**) in contrast, milder CD3+ infiltrated in a patient with mild inflammatory cardiomyopathy (P5); (**F**) CD68+ macrophage infiltrates in a patient with SARS-CoV-2 borderline myocarditis (P2). Scale bars: (**A**): 100 µm; (**B**,**E**,**F**): 50 µm; (**C**,**D**): 200 µm. CD = cluster of differentiation; EBV = Epstein–Barr virus; SARS-CoV2 = severe acute respiratory syndrome coronavirus-2.

A 56-year-old woman with recent history of COVID-19 influential syndrome presented at another institution with oppressive chest pain and anterior ST elevation. Angiography showed normal coronaries, whereas angio-CT ruled out pulmonary embolism and aortic dissection. The echocardiogram showed severe biventricular dysfunction and right ventricular (RV) dilation. The patient was transferred to our ICU on HD inotropes, IABP and empirical iv steroids. However, cardiogenic shock was complicated by multiorgan failure, paroxysmal complete AVB and tachyarrhythmias. The patient was upgraded to venoarterial extracorporeal membrane oxygenator (VA-ECMO) support, and steroid treatment was withdrawn. EMB revealed diffuse acute lymphocytic myocarditis with massive necrosis (Figure 3). Unexpectedly, PCR analysis documented high-load Epstein–Barr virus (2500 copies of DNA/µg). After iv immunoglobulins and progressive weaning from circulatory support, CMR showed LVEF normalization, but persistent RV dilation (97 mL/m^2^) and systolic dysfunction (RVEF 40%) with septal edema. The patient was scheduled for heart transplantation.

## 4. Discussion

### 4.1. Main Findings

We presented a wide spectrum of clinical manifestations of COVID-19-associated myocarditis, in a series of consecutive patients undergoing diagnostic and therapeutic management worked out by a dedicated myocarditis disease unit for myocarditis [4].

### 4.2. Diagnostic Workup

Since myocarditis is often a rule-out diagnosis [6], coronary artery disease was primarily investigated in the majority of our patients. In particular, coronary angiography was performed in cases with cardiogenic shock or clinically suspected myocardial infarction (P5–7), whereas CT scan was mainly used to rule out coronary artery disease in patients with a low pre-test probability (P1,3,4). This is also consistent with the algorithm proposed to investigate myocardial injury in COVID-19 patients [1,3]. In the absence of remarkable T-Tn abnormalities, coronary imaging was spared in a young and low-risk subject (P2).

Among second-level techniques, our strategy aimed at performing both EMB and CMR in the majority of patients (6/8 = 75%). In particular, EMB was needed to define histotype and etiology to allow disease-specific treatment [6]. Of course, EMB was a forced choice in patients with a complicated onset (P4–7, mean LVEF 25 ± 9%), including fulminant and arrhythmic myocarditis [6,7]. However, except for a single case with a normal LVEF and T-Tn normalization (P3), EMB was performed in all patients as the gold standard diagnostic technique [2,6]. Whenever possible, EMB was performed directly at bedside (P1), in compliance with the COVID-19 pandemic logistic needs [8]. As a complementary tool, CMR was chosen both to obtain panoramic heart assessment [5,9], and to allow follow-up disease monitoring (P4,7) [2]. Differently from EMB, CMR was the first exam in clinically stable patients with a normal or mildly reduced LVEF (P1–3, mean LVEF 48 ± 10%) [9,10]. Nonetheless, CMR was performed even in cases with fulminant myocarditis following clinical stabilization (P4,5,7). As a possible alternative to CMR, to avoid ICD-related susceptibility artifacts [11], our findings support the feasibility of FDG-PET scan in patients with arrhythmic myocarditis (P5).

**Figure 4:**
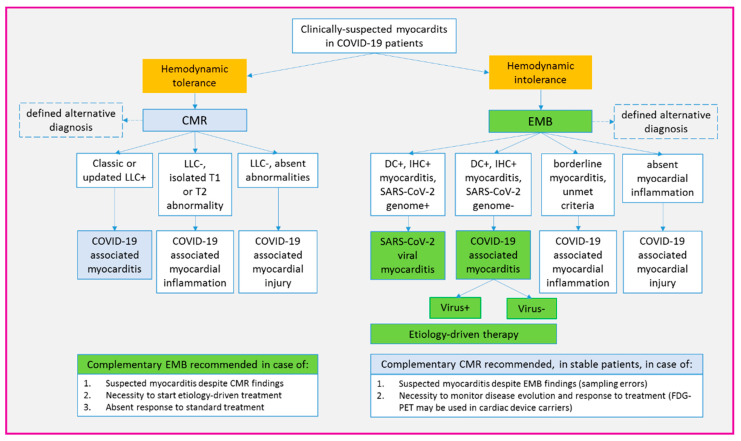
Proposed algorithm for diagnosis and classification of myocarditis in COVID-19 patients. Proposed diagnostic workup for patients with clinically suspected myocarditis associated with COVID-19 infection. The key criterion to guide the initial choice between CMR and EMB in hemodynamic tolerance. However, when feasible, we suggest an extensive use of both techniques, since they provide complementary information. Remarkably, based on the current definition of viral myocarditis (2,6), only EMB allows for SARS-CoV-2 to be identified as responsible for myocardial inflammation. In general, etiology-driven therapy is safe only when myocarditis diagnosis is EMB-proven. CMR = cardiac magnetic resonance; DC = Dallas criteria; EMB = endomyocardial biopsy; FDG-PET= 18F-fluorodeoxyglucose positron emission tomography; IHC = immunohistochemistry; LLC = Lake Louise criteria; SARS-CoV-2 = severe acute respiratory syndrome coronavirus-2.

The overall diagnostic algorithm for COVID-19-associated myocarditis, as suggested by our experience, is summarized in Figure 4.

### 4.3. Disease Features and Etiology

Diagnosis of viral myocarditis was challenging for us in COVID-19 patients, given that, by definition, myocarditis is inflammation and associated myocyte damage in a non-ischemic pattern [3]. Remarkably, the severity of myocardial involvement was often unrelated to the entity of respiratory disease. For instance, P1–3 had pneumonia and non-fulminant myocarditis, whereas P5,7 had fulminant myocarditis in the absence of pneumonia. Furthermore, the timespan between myocarditis and lung disease, as well as between prodromes and cardiac manifestations, ranged from 0 to 12 days (Table 1).

By applying the current definition of EMB-proven myocarditis [6,10], two patients (P1,7) fully met the Dallas criteria, whereas four cases (P2,4–6) had “borderline” inflammation in the absence of necrosis [6,10]. Consistently, although edema was uniformly found (Table 1), the classic LLC were inconstantly met (P3,5,7). Of course, the heterogeneous timespan between EMB and CMR (Table 1) should be acknowledged as a limiting factor. At histology, the most common inflammatory infiltrate was lymphocytic, with a relevant macrophage component in two cases (P2,4). In contrast, interstitial macrophages constituted the dominant infiltration reported in a series of COVID-19 autoptic cases with myocardial inflammation in the absence of clinically suspected myocarditis [12].

Identifying myocarditis etiology was also a major issue in our series, since it provided no direct visualization of SARS-CoV-2 particles within cardiomyocytes. However, in keeping with the definition of viral myocarditis [6], SARS-CoV-2 RNA was isolated from myocardial tissue in one patient (P2): consistently with an N+/ORF1− expression pattern, which has been associated with late-phase or healing infection [13], this patient experienced a favorable clinical course. The importance of PCR analysis is also remarked by the identification of other viral genomes within myocardial samples of three patients. In particular, although a co-pathogenic role cannot be excluded for SARS-CoV-2, we reported unexpected fulminant myocarditis either by Epstein–Barr virus (P7) or PV-B19 (P4). In addition, in keeping with its doubtful pathogenic role [2,14], a low-load and likely bystander PVB-19 genome was documented in a patient (P2) coinfected with SARS-CoV-2. As for virus-negative myocarditis [6], also described in a recent series of COVID-19 autoptic cases [15], the pathophysiology is largely unexplained and deserves further elucidation. A transitory inflammatory stunning following early clearance of the SARS-CoV-2 genome can be hypothesized, given the rapid LVEF recovery documented in many patients despite the absence of IST. However, a role for autoimmunity is suggested in 4/7 AHA-positive cases [16]. The underlying pathophysiology is still to be elucidated. Differential diagnoses may also include takotsubo syndrome as in P1 [17], or preexisting underlying dilated cardiomyopathy as in P4–6, where COVID-19 infection may have just unmasked a preexisting underlying disease.

### 4.4. Arrhythmias and Devices

Although resuscitated sudden cardiac death was observed only in one case (P5), arrhythmias were documented during in-hospital monitoring in a relevant proportion of our cohort (6/8 = 75%). In particular, ventricular arrhythmias were common among ICU patients with fulminant myocarditis (P4–7). As exemplified in Figure 1, it should be noted that ventricular arrhythmias were commonly irregular and polymorphic, hinting at an active inflammatory phase [18]. Among supraventricular arrhythmias, previously reported as the dominant manifestation of clinically stable COVID-19 patients [19], atrial fibrillation was documented only in one patient of our series (P7), in the context of cardiogenic shock. Consistently with the close spatial relationship with the conduction system [20], advanced AVB was documented only in a patient with anteroseptal disease likely unrelated to COVID-19 (P7). As a possible stress-related manifestation [17], a self-limiting episode of an accelerated junctional rhythm was also reported in a case of myocarditis overlapping with a reverse takotsubo pattern (P1).

Concerning the primary prevention of sudden death, our experience supports an ICD-sparing strategy: in fact, most of the patients in our cohort experienced a significant LVEF improvement by the time of hospital discharge (preserved LVEF in 4/7 cases). Dual-chamber ICD was offered to a single case (P5) meeting both secondary and primary prevention criteria for implant: given systolic function normalization and an uneventful 6-month follow-up, however, we acknowledge that a life vest might have been applied instead, as a bridge to decision. In line with the current indications [7], S-ICD was proposed to a patient with a likely preexisting dilated cardiomyopathy and no need for pacing (P6).

### 4.5. Follow-Up and Treatment

In order to reduce the hospital inflow, a multidisciplinary telemedicine platform [4] was applied for follow-up reassessment of clinically stable patients such as P2, as well as home monitoring for all cardiac device carriers [8]. Thus far, no etiology-specific treatment has been identified for COVID-19 patients, either with or without cardiac involvement [2,21]. Nonetheless, a favorable clinical course was observed in the majority of cases, either spontaneously or in response to multimodal treatment including empirical drug regimens to target COVID-19 (Table 2). Remarkably, one patient with viral myocarditis (P4) underwent LVEF improvement after a 3-month treatment course by anakinra: this finding confirms the beneficial effects reported in virus-negative inflammatory cardiomyopathy [22]. In contrast, classic immunosuppression [23,24] is currently contraindicated in patients with viral myocarditis [6]. Accordingly, empirical treatment by iv steroids may have accelerated the evolution towards refractory cardiogenic shock in a patient with a subsequent, unexpected diagnosis of Epstein–Barr myocarditis (P7). Our data support the importance of viral genome screening as a part of the standard assessment of any myocarditis. Additionally, absent mortality in our series suggests a relevant role for circulatory support in patients with cardiogenic shock (P4–7). Regarding EMB-proven virus-negative myocarditis, the importance of specialized immunologic evaluation and ad hoc baseline screening [25] was confirmed by the identification of latent tuberculosis as a contraindication for a safe immunosuppression in a patient (P5). 

### 4.6. Study Limitations

Our data reflect the experience of a single center on a relatively small population. As a result of a patient-tailored strategy, the interval between symptom onset to diagnosis, as well as the timespan between CMR and EMB, was considerably variable; however, this reflects real-world clinical practice. Since in situ hybridization was not performed, the detection of intra-myocardial viruses was hereby limited by PCR sensitivity. Advanced diagnostic and therapeutic tools, as well as the availability of a specialized disease unit for myocarditis, make our experience difficult to be reproduced outside third-level centers. The short follow-up duration is an intrinsic study limitation.

## 5. Conclusions

We presented a patient-tailored strategy for myocarditis associated with COVID-19 infection, as assessed by a dedicated multidisciplinary disease unit at a referral center. Myocarditis diagnosis was achieved following extensive use of both multimodal imaging and EMB, where molecular testing for viral genomes had a key role in the subsequent therapeutic choices. Although myocarditis was associated with recent-onset COVID-19 infection, alternative etiologies beyond SARS-CoV-2 were frequently documented. Our findings suggest that a hub-and-spoke model can be extensively applied during the current COVID-19 pandemic, in order to offer advanced and personalized workup, especially for patients with complicated myocarditis. Meanwhile, multicenter prospective registries are called to fill the knowledge gaps concerning characterization and outcomes of COVID-19-associated myocardial inflammatory syndromes.

## Figures and Tables

**Table 1:** Baseline features and diagnostic workup.

Features	P1	P2	P3	P4	P5	P6	P7
General features							
Age, y	43	38	58	45	64	50	56
Gender	female	male	female	male	male	male	female
Ethnicity	African American	African American	Caucasian	Caucasian	Caucasian	Caucasian	Caucasian
COVID diagnosis	NPS	NPS	NPS	NPS, BAS, BAL	NPS	NPS	NPS
Pneumonia diagnosis	XR, CT	XR, CT	XR, CT	XR, CT	negative	XR, CT	negative
Delay from first cardiac abnormality to pneumonia, days	0	6	0	8	no	12	no
Clinical presentation							
Myocarditis presentation	ACS-like	HF	ACS-like	HF	VA	HF	ACS-like, HF
Sp02, %	89	96	96	88	81	88	76
T, °C	37.7	36.5	37.0	38.5	38.7	37.1	36.2
Prodromes	fever, vomit	cough	fever, vomit, diarrhea	fever, vomit, diarrhea	no	cough	fever
Delay from prodromes to cardiac symptoms, days	2	7	7	5	0	10	12
Coronary artery assessment	CT	no	CT	CT	CA	CA	CA
Blood exams							
WBC peak, 10^6^/ml	18.9	8.7	4.4	19.8	28.6	9.0	28.7
CRP peak, mg/L	21	208	52	309	233	12	79
T-Tn peak, ng/L	135	26	222	39	487	29	13,722
NTproBNP peak, pg/mL	1001	148	261	24,252	1170	1122	15,131
Screening for autoimmunity	AHA, anti-TPO	AHA	normal	ACL IgG	AHA, ANCA	normal	AHA
Electrocardiogram (ECG) and arrhythmias							
Presentation rhythm	atrial ectopic	sinus	sinus	sinus	VF	sinus	sinus
PQ, ms	170	159	147	172	167	216	192
QRS, ms	94	90	82	98	82	105	130
QTc, md	452	444	392	378	421	456	445
Abnormal ST	yes	no	yes	no	yes	no	yes
Abnormal T-waves	yes	yes	yes	no	yes	yes	yes
BA	AJR (self-limited)	no	no	-	-	1st-degree AVB	3rd-degree AVB, LBBB
SVA	no	no	no	-	-	-	AF
VA	no	no	no	PVC, NSVT	VF, NSVT, PVC	PVC, NSVT	NSVT, VT
Echocardiogram and CMR							
LVEDVi, mL/m^2^	52	61	47	77	82	115	44
LVEF, %	43	41	59	34	15	20	30
Dominant WMA localization	IL, reverse TTS	AS	no	diffuse	diffuse	IL	AS
TAPSE, mm	18	18	22	12	14	23	8
RVEDD, mm	27	26	25	38	32	40	50
CMR timing, day	7	8	7	99	13	-	17
LGE	no	no	mid-basal AS (subepicardial)	0	mid-basal IL (subepicardial)	-	basal septal (midwall), RV
STIR	mid-basal diffuse	diffuse basal, mid septal	AS	diffuse	mid-basal IL	-	mid-basal septal, RV
T1 max, ms	1234	1125	1311	1055	1159	-	1232
T2 max, ms	67	58	62	54	67	-	65
Pericardial effusion	No	mild	no	mild	mild	-	mild
EMB							
EMB timing, day	7	9	-	17	9	118	7
Edema	Yes	yes	-	yes	yes	yes	yes
Inflammatory infiltrates	yes	yes	-	yes	yes	yes	yes
CD3+ > 7/mm^2^	yes	no	-	yes	yes	yes	yes
CD68+ > 4/mm^2^	no	yes	-	yes	no	no	no
Necrosis	mild	no	-	no	no	no	massive
Replacement fibrosis	no	mild	-	mild	no	mild	no
Viral genome	no	PVB19 (low-load), SARS-CoV2 (N+/ORF1−)	-	PVB19 (high-load)	no	no	EBV (high-load)

Complete features of patients (P1–P7) and baseline diagnostic workup are shown. ACL = anti-cardiolipin; ACS = acute coronary syndrome; AF = atrial fibrillation; AHA = anti-heart autoantibodies; AJR = accelerated junctional rhythm; ANCA= anti-neutrophil cytoplasmatic autoantibodies; AS = anteroseptal; AVB = atrioventricular block; BA = bradyarrhythmias; BAL = bronchoalveolar lavage; BAS = bronchoaspirate; CA = coronary angiography; CD = cluster of differentiation; CMR = cardiac magnetic resonance; CRP = C-reactive protein (n.v. < 6 mg/L); CT = computed tomography; EBV = Epstein–Barr virus; EMB = endomyocardial biopsy; HF = heart failure; IL = inferolateral; LBBB = left bundle branch block; LGE = late gadolinium enhancement; LVEDVi = left ventricular end-diastolic volume (indexed); LVEF = left ventricular ejection fraction; NPS = nasopharyngeal swab; NSVT = nonsustained ventricular tachycardia; NTproBNP = N-terminal pro-brain natriuretic pepetide (n.v. < 88 pg/mL); PVB19 = parvovirus B19; PVC = premature ventricular complexes; RVEDD = right ventricular end-diastolic diameter (RV2); SARS-CoV2 = severe acute respiratory syndrome coronavirus-2 (N and ORF1 genes); STIR = short-tau inversion recovery; SVA = supraventricular arrhythmias; T = temperature; T-Tn = T-troponin (n.v. < 14 ng/L); T1/T2 = T-mapping sequences (T1 n.v. < 1045 ms; T2 n.v.< 50 ms); TAPSE = tricuspid annular plane systolic excursion; TPO = tyroid peroxydase; VA = ventricular arrhythmias; VF = ventricular fibrillation; VT = ventricular tachycardia; WBC = white blood cells; WMA = wall motion abnormalities; XR = X-ray.

**Table 2:** Treatment and follow-up.

Features	P1	P2	P3	P4	P5	P6	P7
Acute-phase treatment							
ICU stay, days	0	0	2	7	5	0	24
Iv diuretics	yes	yes	no	yes	yes	yes	yes
Inotropes	no	no	no	Adr, Nor, Lev	Adr, Nor	dopamine	Adr, Nor, Lev
MCS	no	no	no	IABP	no	no	IABP, VA-ECMO
Mechanical ventilation	no	no	no	yes	no	no	yes
Cardiac devices	no	no	no	no	ICD	S-ICD	temporary PM
Etiology-driven treatment	HCQ, L/R	AZT	HCQ	HCQ, AKR	no	HCQ, AZT, AKR, PDN + AZA	PDN, IVIG
Discharge assessment							
Hospitalization, days	13	35	11	31	19	36	24
Symptoms	no	no	mild chest pain	no	dyspnea	dyspnea	dyspnea
NYHA class	I	I	I	II	II	III	II
T-Tn, ng/L	11	10	5	13	24	8	1416
NTproBNP, pg/ml	76	136	261	121	358	383	1937
LVEF, %	65	61	60	45	40	20	65
TAPSE, mm	20	22	24	16	18	20	10
RAAS inhibitors	no	no	no	ramipril	enalapril	valsartan	no
Betablockers	no	no	bisoprolol	metoprolol	metoprolol	bisoprolol	no
Antiarrhythmics	no	no	no	no	no	AMD	ivabradin
Diuretics	no	no	SL	FS/SL	FS/SL	FS/SL	FS/CR
Follow-up assessment							
Last follow-up, months	9	6	6	6	6	6	3
Follow-up mode	IP, TM	TM	IP, TM	IP	IP, TM	IP, TM	TM
Death	no	no	no	no	no	no	no
Arrhythmias	no	no	no	no	PVC	PVC, NSVT	PVC, NSVT, AF
End-stage heart failure	no	no	no	no	no	referred for HTx	referred for HTx
New hospitalization	no	no	no	no	no	no	no
Symptoms	no	no	dyspnea on effort	no	no	dyspnea	dyspnea
NYHA class	I	I	II	I	I	III	II
T-Tn, ng/L	5	6	6	8	13	6	32
NTproBNP, pg/ml	56	88	154	76	103	272	887
LVEF, %	64	63	61	47	60	24	63
TAPSE, mm	20	21	25	25	20	20	12

Tailored treatment strategies and outcomes are shown for all patients (P1–P7). AF = atrial fibrillation; Adr = adrenaline; AKR = anakinra; AMD = amiodarone; AZA = azathioprine; AZT = azithromycine; CR = canrenoate; FS = furosemide; HCQ = hydroxychloroquine; HTx = heart transplantation; IABP = intra-aortic balloon pump; ICD = implantable cardioverter defibrillator; ICU = intensive care unit; IP = in-person; IVIG = intravenous immunoglobulins; L/R = lopinavir/ritonavir; Lev = levosimendan; LVEF = left ventricular ejection fraction; MCS = mechanical circulatory support; Nor = noradrenaline; NSVT = nonsustained ventricular tachycardia; NTproBNP = N-terminal pro-brain natriuretic pepetide (n.v. < 88 pg/mL); NYHA = New York Heart Association; PDN = prednisone; PM = pacemaker; PVC = premature ventricular complexes; RAAS = renin–angiotensin–aldosterone system; S-ICD = subcutaneous ICD; SL = spironolactone; T-Tn = T-troponin (n.v. < 14 ng/L); TM = telemedicine; TAPSE = tricuspid annular plane systolic excursion; VA-ECMO = venoarterial extracorporeal membrane oxygenator.

## Data Availability

Data will be made available, upon reasonable request, by emailing the correspondent author.

## Supplementary Materials

The following are available online at https://www.mdpi.com/article/10.3390/jcm10091974/s1, Online Supplements.

## Abbreviations

AHA = anti-heart autoantibodies; AVB = atrioventricular block; CMR = cardiac magnetic resonance; CT = computed tomography; EMB = endomyocardial biopsy; FDG-PET = 18F-fluorodeoxyglucose positron emission tomography; IABP= intra-aortic balloon pump; ICD = implantable cardioverter defibrillator; ICU = intensive care unit; LLC = Lake Louise criteria; LV = left ventricular; LVEF = left ventricular ejection fraction; NPS = nasopharyngeal swab; NSVT = nonsustained ventricular tachycardia; PV-B19 = parvovirus B19; PVCs = premature ventricular complexes; RAAS = renin–angiotensin–aldosterone system; RV = right ventricular; SARS-CoV2 = severe acute respiratory syndrome coronavirus-2; T-Tn= T-troponin; VA-ECMO = venoarterial extracorporeal membrane oxygenator; VT = ventricular tachycardia.
