# Supplementary material for: The Spectrum of COVID-19-Associated Myocarditis: A Patient-Tailored Multidisciplinary Approach
Source: J Clin Med. 2021 May 4;10(9):1974. doi: 10.3390/jcm10091974 (PMC8124580; doi:10.3390/jcm10091974)
Supplement: Supplementary file 1 [file jcm-10-01974-s001.zip › jcm-1167232-supplementary.pdf]

The spectrum of COVID-19-associated myocarditis: a patient-tailored multidisciplinary approach – Online Supplements

SUPPLEMENTARY METHODS

Inclusion criteria

The study protocol conforms to the ethical guidelines of the 1975 Declaration of Helsinki. After approval by the Institutional Review Board, enrolled patients gave written informed consent for study participation. The following inclusion criteria apply to this study: 1) age ≥ 18 years; 2) newly-diagnosed COVID-19 infection; 3) hospitalization with new onset cardiac symptoms, including syncope, palpitation, dyspnea or chest pain and clinically suspected myocarditis, based on the 2013 European Society of Cardiology position paper on myocarditis (1); 4) final diagnosis of myocarditis by cardiac magnetic resonance (CMR) and/or endomyocardial biopsy (EMB), based on accepted criteria (see below).

Baseline patient workup

Standard cardiological evaluation for clinically suspected myocarditis consisted of 12-lead ECG, continuous ECG telemonitoring, transthoracic echocardiogram, and blood exams including C-reactive protein, erythrocyte sedimentation rate, high sensitivity cardiac T-troponin (T-Tn) and NTproBNP. Coronary angiography or CT scan were performed to rule out epicardial coronary artery disease (1).

COVID-19 respiratory infection

Diagnosis of COVID-19 respiratory infection (2,3) was made by nasopharyngeal swab (NPS), associated with either bronchoaspirate (BAS) or bronchoalveolar lavage (BAL) in selected cases. NPS diagnosis was accepted only following molecular testing (rapid antigenic testing excluded) confirmed by at least double assessment. Chest X-rays (CXR) or computerized tomography (CT) allowed diagnosis of COVID-19 interstitial pneumonia.

Endomyocardial biopsy

Right ventricular EMB was performed in all of the patients, via right internal jugular vein ascess. A Cordis® bioptome, 7 Fr sheath, was used. Under fluoroscopic and echocardiographic guidance, sampling was performed at interventricular septum. Five samples of endomyocardial tissue per patient were obtained. To comply with COVID-19-related preacutions (4), as well as for urgent cases, EMB was performed directly at bedside.

Histology, immunohistochemistry, and molecular analysis

Myocardial tissue was analysed by histology, immunohistochemistry, and molecular biology, at an external referral center for Cardiovascular Pathology. In detail, 4 tissue samples were immediately fixed in 10% buffered formalin at room temperature for light microscopy and immunohistochemistry, and 1 sample was stored in RNA later tubes at room temperature for viral polymerase chain reaction (PCR). Samples were analyzed by two experienced cardiac pathologists, and results were independently confimred. EMB-proven myocarditis was defined, as currently accepted (1,5), by histological, immunohistochemical and molecular criteria. In detail: a) histology: Dallas criteria, defined as histological evidence of inflammatory infiltrates within the myocardium associated with myocyte degeneration and necrosis of nonischaemic origin; however, we also included patients with no evidence of necrosis, but significant inflammatory infiltrates ("borderline" myocarditis); in the presence of borderline myocarditis, patients were enrolled only if all the remaining criteria for myocarditis were met at EMB, consistently with clinical and CMR findings (1); b) immunohistochemistry: presence of an abnormal inflammatory infiltrate, defined as follows: ≥ 14 leucocytes/mm^2 including up to 4 monocytes/mm^2 with the presence of CD 3 positive T-lymphocytes ≥ 7 cells/mm^2; c) molecular biology: classification into viral forms, defined as histological evidence for myocarditis associated with positive viral PCR; and autoimmune or virus-negative myocarditis, defined as histological myocarditis with negative viral PCR. Based on epidemiology, in the absence of clinical or

laboratory findings suggesting other infective etiologies, a standard panel of viruses was analyzed, including: SARS-CoV-2, coxsackieviruses A and B, echoviruses, rhinoviruses, influenza A and B viruses, adenoviruses, parvovirus B19, cytomegalovirus, human herpes virus-6, Epstein-Barr virus, varicella-zoster virus, herpes simplex virus-1. In the case of viral myocarditis, PCR was performed on blood sample too, to evaluate for coexisting viremia. Viral myocarditis was deemed in the presence of detectable umbalance between intramyocardial viral load (high) and viremia (lower) (1,5). For SARS-COV-2-proven myocarditis, molecular analysis included the sequencing of the ORF1 and N genes by reverse-trascriptase (RT) PCR (3,6).

Cardiac magnetic resonance

CMR was performed on a 1.5 T scanner (Achieva dStream; Philips Medical Systems, Eindhoven, The Netherlands) equipped with a 32-channel phased-array coil (voxel size 1.4 × 1.4 mm, slice thickness 3.5 mm). Images were acquired accoriding to the updated Society for Cardiac Magnetic Resonance recommendations (7), implemented with the evaluation of standard and updated Lake Louise criteria (8,9). Functional imaging consisted of ECG-gated balanced steady state free precession cine sequences acquired during breath hold in horizontal and vertical long axis, left ventricular (LV) outflow tract and short axis, the latter covering the whole LV from base to apex for the assessment of volumes and function. Biventricular indexed end-diastolic volume (EDVi) and EF were calculated by the cardiac software after drawing the endocardial and epicardial borders in the end-diastolic and end-systolic short-axis cine-SSFP images. Cutoffs for chamber dilatation and systolic dysfunction in CMR were referred to updated international standards (10). Myocardial inflammation and edema was evaluated using black blood T2- short tau inversion recovery (STIR) images in horizontal and vertical long axis, LV outflow tract and short axis, the latter covering the whole LV from base to apex, native-T1 mapping and T2 mapping, both acquired in the short axis plane (base, mid-ventricle, apex). Modified Look-Locker inversion recovery sequences and gradient-(echo planar imaging) and spin-echo multi-echo sequences were used for T1-mapping and T2-mapping, respecitively. Myocardial hyperemia was evaluated using early gadolinium enhancement (EGE) and early T1 shortening (eT1sh): native-T1 map and early-enhanced T1 map (2 min after injection of 0.15mmol/kg of gadobutrol [Gadovist; Bayer Healtare, Berlin, Germany]) acquired in the three matched short axis planes (base, mid-ventricle, apex) were precessed according to the following formula: eT11sh = -[(earlyT1myocardium - nativeT1myocardium)/native T1myocardium] (11). Late gadolinium enhancement (LGE) images were acquired 10 min after gadolinium injection using 2D T1 weighted segmented inversion-recovery gradient-echo sequences acquired in horizontal, vertical long axis, and short axis, the latter covering the whole LV. The correct inversion time was determined using the Look-Locker technique. All CMR examinations were centrally analyzed by using a dedicated semi-automatic software (CVI42 v.5.6.6, Circle Cardiovascular Imaging, Calgary, Canada), and results were independently confirmed by three experienced observers.

FDG-PET scan

Preparation to ^{18}F-Fluorodeoxyglucose positron emission tomography (FDG-PET) consisted of a low-carbohydrate-high-fat diet for 12 hours, followed by a 12-hour fast, in order to achieve suppression of physiologic glucose myocardial metabolism (12). Glucose blood level evaluation was followed by the injection of a standard dose (3.5-4.5 MBq/kg) of ^{18}F-FDG via antecubital vein. Thorax cardiac gated PET scan was acquired at approximately 60 minutes, by FDG-PET/computed tomography (CT) scanner GE Healthcare. FDG-PET/CT acquisition protocol consisted of X-ray scout view to define anatomy, followed by low-dose whole-body CT scan (kVp=120, 30-40mA, Cine Mode), and PET scan (20 min, List Mode). Post-processing included both static and gated mode reconstructions, performed using a 3D algorithm integrating all the available models (TOF, PSF, Regularization). Parameters were optimized to obtain the best image quality, and 8-10 bins were used to perform gated mode reconstruction. FDG-PET positivity was defined in the presence of focal pathological FDG uptake, as assessed indepentently by two experienced readers. FDG-PET was used instead of CMR in selected patients carrying implantable cardioverter defibrillator (ICD), to avoid susceptibility artifacts (13).

Cardiac autoantibodies

Serum samples from myocarditis patients were tested for cardiac autoantibodies. In particular, to determine anti-heart autoanditbodies (AHA), indirect immunofluorescence assay was used, according to defined protocols (14,15). For each assay, two serum samples were used as standard positive and negative controls.

Ventricular arrhythmia characterization

For ventricular arrhythmias (VA), morphology was defined by 12-lead ECG analysis, as previously described (16). For both sustained (> 3 consecutive complexes originating in the ventricles at a rate > 100 bpm, lasting > 30 s or requiring termination due to hemodynamic compromise) and nonsustained (spontaneously terminating within 30 s) ventricular tachycardias (17), the cutoff for cyclye length irregularity was 40 ms (16,18). Ventricular fibrillation was defined as irregular electrical activity with ventricular rate > 300 bpm (17).

Multidisciplinary disease unit

As previously described (19), myocarditis disease unit (MDU) is multidisciplinary team composed by cardiac electrophysiologists, clinical cardiologists, radiologists with special expertize in CMR, nuclear medicine specialists, cardiac pathologists, dedicated immunologists, infectious disease specialists, and geneticians. In patients with fulminant myocarditis (20), the team was extended to include intensive care unit specialists as key players for the acute-phase assistance and circulatory support.

General treatment

Circulatory support for fulminant myocarditis consisted of inotropes, intra-aortic balloon pump, Impella and venoarterial extracorporeal membrane oxygenator, with a patient-tailored indication (21,22). Cardiological medical treatment, including renin-angiotenin-aldosterone-system inhibitors, diuretics and betablockers, was patient-tailored. In turn, antiarrhythmic therapy, as well as implant of cardiac devices, was upon clinical indication, integrating international guidelines recommendations (23,24), and the experience of a tertiary level center for ventricular arrhythmia management (16,25). Treatment for COVID-19 infection was empirical, reflecting the evolution of medical knowledge from the pandemic outbreak onset (26) to the current state of the art (5,27).

Immunosuppressive therapy

Based on updated recommendations (1,5), when indicated, accepted and non-contraindicated, patients received immunosuppressive therapy (IST). Criteria for IST eligibility included all of the following: 1) EMB-proved diagnosis of active myocarditis; 2) absence of pathogenic viral genome in the myocardium; 3) absence of clinical contraindications to immunosuppression; 4) patient acceptance. Consistently with the TIMIC Trial results (28) and current recommendations (1,5), first choice treatment consisted of a double drug regimen, including 1 mg/kg prednisone daily and 2 mg/kg azathioprine daily. Alternative immunosuppressive agents were used (29), based on clinical indication, as assessed by an experienced team of cardiac immunologists. Baseline screening to rule-out contraindications to IST, as well as strict follow-up schedule to rule-out treatment toxicity, have been previously described (15).

Follow-up

In all patients, follow-up reassessment included standard ECG, 12-lead 24-h Holter ECG monitoring, device interrogation when appropriate, echocardiographic, laboratory exams including cardiac (T-troponin, NTproBNP) and inflammatory biomarkers (C-reactive protein, erhythrocyte sedimentation rate), and second-level imaging techniques (CMR, FDG-PET), based on clinical indication. For patients with active-phase myocarditis, the local protocol (15,16) include minimum 4 reassessments per year during the first year, and 2 reassessments per year later. Consistently, in our series follow-up visits were obtained at month 1, 3, 6, and 9. For either symptomatic or clinically-unstable patients, in-person follow-up was planned despite the restrictions related to the COVID-19 pandemic (4). Instead, telemedicine was applied to clinically-stable patients, by using a dedicated multidisciplinary HTML5 web platform (HealthMeeting®, by Wezen Technologies s.r.l.), as previously described (19). Furthermore, all cardiac device carriers underwent continuous home-monitoring for arrhythmias (4).

Endpoints

Study endpoints were assessed by hospital discharge (primary endpoints) and during follow-up (secondary endpoints) and included: 1) occurrence of cardiac death; 2) occurrence of arrhythmias, including sustained/nonsustained ventricular tachycardia, ventricular fibrillation, appropriate anti-tachycardia pacing or shock in ICD carriers, atrial fibrillation, $2^{nd}/3^{rd}$-degree atrioventricular blocks, and Lown's grade ≥ 2 premature ventricular complexes (30); 3) occurrence of end-stage heart failure supporting indication to heart transplantation; 4) occurrence of new hospitalizations for acute heart failure; 5) occurrence or persistence of caridiac symptoms (chest pain, dyspnea, palpitation, syncope); 6) worsening of New York Heart Association class; 7) persistent abnormalities in T-troponin and NTproBNP values; 8) abnormal LV ejection fraction (LVEF < 50%) or trsicuspid annular plane systolic excursion (TAPSE < 20 mm). All the above cited endpoints are assessed as part of a defined protocol (15,16).

Statistical analysis

Continuous variables were expressed as means and standard deviations or as medians and IQRs of 25th to 75h percentiles, depending on the data distribution. Categorical variables were reported as counts and percentages.

SUPPLEMENTARY REFERENCES

1. Caforio AL, Pankuweit S, Arbustini E, et al. European Society of Cardiology Working Group on Myocardial and Pericardial Diseases. Current state of knowledge on aetiology, diagnosis, management, and therapy of myocarditis: a position statement of the European Society of Cardiology Working Group on Myocardial and Pericardial Diseases. Eur Heart J 2013;34:2636-2648.
2. Rehman MFU, Fariha C, Anwar A, et al. Novel coronavirus disease (COVID-19) pandemic: A recent mini review. Comput Struct Biotechnol J. 2020 Dec 31.
3. Chu DKW, Pan Y, Cheng SMS, et al. Molecular Diagnosis of a Novel Coronavirus (2019-nCoV) Causing an Outbreak of Pneumonia. Clin Chem. 2020;66:549-555.
4. Mazzone P, Peretto G, Radinovic A, et al. The COVID-19 challenge to cardiac electrophysiologists: optimizing resources at a referral center. J Interv Card Electrophysiol. 2020;18:1-7.
5. Tschöpe C, Ammirati E, Bozkurt B. Myocarditis and inflammatory cardiomyopathy: current evidence and future directions. Nat Rev Cardiol. 2020:1-25.
6. Baggiano A, Rizzo S, Basso C, Pontone G. A patient with rapid worsening dyspnoea during Covid-19 pandemic. Eur Heart J. 2020 Dec 11:ehaa988.
7. Kramer CM, Barkhausen J, Flamm SD, et al.; Society for Cardiovascular Magnetic Resonance Board of Trustees Task Force on Standardized Protocols. Standardized cardiovascular magnetic resonance (CMR) protocols 2013 update. J Cardiovasc Magn Reson. 2013 Oct 8;15:91. doi: 10.1186/1532-429X-15-91.
8. Friedrich MG, Sechtem U, Schulz-Menger J, et al.; International Consensus Group on Cardiovascular Magnetic Resonance in Myocarditis. Cardiovascular magnetic resonance in myocarditis: A JACC White Paper. J Am Coll Cardiol 2009;53:1475-87.
9. Ferreira VM, Schulz-Menger J, Holmvang G, at al. Cardiovascular Magnetic Resonance in Nonischemic Myocardial Inflammation: Expert Recommendations. J Am Coll Cardiol. 2018;72::3158-3176.
10. Kawel-Boehm N, Maceira A, Valsangiacomo-Buechel ER, et al. Normal values for cardiovascular magnetic resonance in adults and children. J Cardiovasc Magn Reson 2015;18:17-29.
11. Palmisano A, Benedetti G, Faletti R, et al. Early T1 Myocardial MRI Mapping: Value in Detecting Myocardial Hyperemia in Acute Myocarditis. Radiology. 2020 Mar 10:191623

12. Manabe O, Yoshinaga K, Ohira H, et al. The effects of 18-h fasting with low-carbohydrate diet preparation on suppressed physiological myocardial (18)F-fluorodeoxyglucose (FDG) uptake and possible minimal effects of unfractionated heparin use in patients with suspected cardiac involvement sarcoidosis. J Nucl Cardiol. 2016 Apr;23(2):244-52.
13. Palmisano A, Vignale D, Peretto G, et al. Hybrid FDG-PET/MR or FDG-PET/CT to Detect Disease Activity in Patients With Persisting Arrhythmias After Myocarditis. JACC Cardiovasc Imaging. 2020; 10:S1936-878X(20)30266-7.
14. Caforio A, Mahon Ng, Baig Mk, et al. Prospective familial assessment in dilated cardiomyopathy - Cardiac autoantibodies predict disease development in asymptomatic relatives. Circulation, 2007 vol. 115, p. 76-83.
15. Peretto G, Sala S, De Luca G, et al. Immunosuppressive Therapy and Risk Stratification of Patients With Myocarditis Presenting With Ventricular Arrhythmias. JACC Clin Electrophysiol. 2020;6:1221-1234.
16. Peretto G, Sala S, Rizzo S, et al. Ventricular Arrhythmias in Myocarditis: Characterization and Relationships With Myocardial Inflammation. J Am Coll Cardiol. 2020;75:1046-1057.
17. Cronin EM, Bogun FM, Maury P, et al. 2019 HRS/EHRA/APHRS/LAHRS expert consensus statement on catheter ablation of ventricular arrhythmias. J Arrhythm. 2019;35:323-484.
18. Oreto G, Satullo G, Luzza F, Donato A, Scimone IM, Cavalli A. Irregular ventricular tachycardia: a possible manifestation of longitudinal dissociation within the reentry pathway. Am Heart J 1992;124:1506-1511.
19. Peretto G, De Luca G, Campochiaro C, et al. Telemedicine in myocarditis: Evolution of a mutidisciplinary "disease unit" at the time of COVID-19 pandemic. Am Heart J. 2020;229:121-126.
20. Ammirati E, Frigerio M, Adler ED, et al. Management of Acute Myocarditis and Chronic Inflammatory Cardiomyopathy: An Expert Consensus Document. Circ Heart Fail. 2020;13:e007405.
21. Saeed D, Potapov E, Loforte A, et al. Transition From Temporary to Durable Circulatory Support Systems. J Am Coll Cardiol. 2020;76:2956-2964.
22. Spillmann F, Van Linthout S, Schmidt G et al. Mode-of-action of the PROPELLA concept in fulminant myocarditis. Eur. Heart J. 2019;40:2164-2169.
23. Priori SG, Blomström-Lundqvist C, Mazzanti A, at al. Task Force for the Management of Patients with Ventricular Arrhythmias and the Prevention of Sudden Cardiac Death of the European Society of Cardiology (ESC). 2015 ESC Guidelines for the management of patients with ventricular arrhythmias and the prevention of sudden cardiac death. Europace 2015;17:1601-1687.
24. Al-Khatib SM, Stevenson WG, Ackerman MJ, at al. 2017 AHA/ACC/HRS guideline for management of patients with ventricular arrhythmias and the prevention of sudden cardiac death. Heart Rhythm 2018;15:e190-e252.
25. Diagnostic and therapeutic approach to myocarditis patients presenting with arrhythmias. Peretto G, Sala S, Della Bella P. G Ital Cardiol. 2020;21:187-194.
26. Lim J, Jeon S, Shin HY, et al. Case of the Index Patient Who Caused Tertiary Transmission of COVID-19 Infection in Korea: the Application of Lopinavir/Ritonavir for the Treatment of COVID-19 Infected Pneumonia Monitored by Quantitative RT-PCR. J Korean Med Sci. 2020 Feb 17;35(6):e79.
27. Sanders JM, Monogue ML, Jodlowski TZ, Cutrell JB. Pharmacologic treatments for coronavirus disease 2019 (COVID-19): a review. JAMA. 2020;323:1824-1836.
28. Frustaci A, Russo MA, Chimenti C. Randomized study on the efficacy of immunosuppressive therapy in patients with virus-negative inflammatory cardiomyopathy: the TIMIC study. Eur Heart J. 2009;30:1995-2002.
29. De Luca G, Campochiaro C, Sartorelli S, et al. Therapeutic strategies for virus-negative myocarditis: a comprehensive review. Eur J Intern Med 2020;S0953-6205(20)30170-9.

30. Lown B, Wolf M. Approaches to sudden death from coronary heart disease. Circulation 1971;44:130-142.
